# Long-Term Effect of Outdoor Air Pollution on Mortality and Morbidity: A 12-Year Follow-Up Study for Metropolitan France

**DOI:** 10.3390/ijerph15112487

**Published:** 2018-11-08

**Authors:** Shreosi Sanyal, Thierry Rochereau, Cara Nichole Maesano, Laure Com-Ruelle, Isabella Annesi-Maesano

**Affiliations:** 1 Epidemiology of Allergic and Respiratory Diseases Department, IPLESP, Medical School Saint-Antoine, Sorbonne Université and INSERM, F75012 Paris, France; shreosi.sanyal@iplesp.upmc.fr (S.S.); cara.maesano@iplesp.upmc.fr (C.M.); 2 Institute for Research and Documentation in Health Economics, F75019 Paris, France; Rochereau@irdes.fr (T.R.); Comruelle@irdes.fr (L.C.-R.)

**Keywords:** air pollution, morbidity, mortality, respiratory diseases, cardiovascular diseases

## Abstract

Background: Short-term effects of air pollution are documented more than long-term effects. Objective: We investigated 12-year impacts of ambient air pollutants on cardiovascular and respiratory morbidity and mortality at the departmental level in metropolitan France. Methods: Daily air pollution data at 2-km resolution, including concentrations of particulate matter of 10 µm or 2.5 µm in diameter or less (PM_10_ and PM_2.5_), nitrogen dioxide (NO_2_), and ozone (O_3_), were accrued from the CHIMERE database for 1999 and 2000. Simultaneously, morbidity (hospitalizations) and mortality data were collected in 2012 using the ESPS (Enquête Santé et Protection Sociale/Health, Health Care and Insurance Survey) survey data and the CepiDc (Centre d’Épidémiologie sur les Causes Médicales de Décès/French Epidemiology Centre on Medical Causes of Death) database. Based on Poisson regression analyses, the long-term effect was estimated. A higher risk of all-cause mortality was observed using CépiDc database, with a relative risk of 1.024 (95% CI: 1.022, 1.026) and 1.029 (95% CI: 1.027, 1.031) for a 10 µg/m^3^ increase in PM_2.5_ and PM_10_, respectively. Mortality due to cardiovascular and respiratory diseases likewise exhibited long-term associations with both PM_2.5_ and PM_10_. Using ESPS survey data, a significant risk was observed for both PM_2.5_ and PM_10_ in all-cause mortality and all-cause morbidity. Although a risk for higher all-cause mortality and morbidity was also present for NO_2_, the cause-specific relative risk due to NO_2_ was found to be lesser, as compared to PM. Nevertheless, cardiovascular and respiratory morbidity were related to NO_2_, along with PM_2.5_ and PM_10_. However, the health effect of O_3_ was seen to be substantially lower in comparison to the other pollutants. Conclusion: Our study confirmed that PM has a long-term impact on mortality and morbidity. Exposure to NO_2_ and O_3_ could also lead to increased health risks.

## 1. Introduction

Air pollution is a major risk factor for human health and in particular for respiratory and cardiovascular morbidity and mortality [1,2]. Air pollutants can cause both short- and long-term health effects in human beings, and the exposure related health impact varies for each pollutant [3]. It is common to see exacerbations in chronic respiratory diseases like bronchitis, COPD, asthma, and reduced lung function due to exposure to various ambient air pollutants [2]. Particulate matter (PM) and other air pollutants are found to be closely associated with long-term effects on cardiovascular diseases [4,5,6] as well as respiratory diseases [7] in both clinical and epidemiological studies. In addition to PM, NO_2_ was also related to long-term effects [8,9], whereas fewer studies exist for O_3_ [10].

These long-term morbidity effects can eventually aggravate and cause mortality. Some cohort-based studies have indicated long-term effects of air pollutants on mortality [11,12]. A study [13] on continental France ascertained that 9% of total mortality in France is due to anthropogenic PM_2.5_. A Health Impact Assessment (HIA) study from 1996 estimated that 31,700 and 17,600 attributable cases of long-term mortality were observed for French adults above 30 years of age, in the case of PM_2.5_ and PM_10_ exposure, respectively [14]. However, other atmospheric pollutants can also cause health hazards leading to mortality. 

In our present study, we evaluated the long-term association of the major air pollutants (PM_10_, PM_2.5_, NO_2_, O_3_) with morbidity and mortality in metropolitan France, based on pre-existing data. A multipollutant approach was used for this purpose, which is based on daily fine scale assessments of air pollutant levels.

## 2. Materials and Methods 

We used existing datasets of both air pollution and health outcomes at the metropolitan French level (referring to the European part of France).

### 2.1. Air Pollution Data Sources 

The concentrations of air pollutants were obtained through the CHIMERE chemistry transport model for Metropolitan France [15]. In the present study, the model is based on annual average concentrations of air pollutants with a 2-km resolution for 1999–2000 after the refinement of mesh and assimilation of daily data. A geostatistical analysis was carried out for estimating the concentration of air pollutants. However, the air pollutant concentrations were pooled and up-scaled to each of the 96 departments (territorial collectivities) of mainland France for our subsequent analyses. Average concentrations (µg/m^3^) at the department level were considered in the models. 

### 2.2. Health Outcomes Assessment

Health outcomes of interest included morbidity and mortality, taking into consideration natural causes as well as some specific causes for the year 2012. Two sources were used for obtaining health outcome data, which comprised of:

1. National routine statistics of mortality as provided by the CépiDc (Centre d’Épidémiologie sur les Causes Médicales de Décès/French Epidemiology Centre on Medical Causes of Death) of INSERM (Institut National de la Santé et de la Recherche Médicale) (http://www.cepidc.inserm.fr/).
2. The National ESPS survey **(**Enquête Santé et Protection Sociale/Health, Health Care and Insurance Survey) of IRDES (Institut de Recherche et Documentation en Économie de la Santé/Institute for Research and Documentation in Health Economics), which was conducted using a random sample in Metropolitan France and is representative of the general population living there [http://www.irdes.fr/english/french-surveys/esps-health-health-care-and-insurance survey.html]. For the purpose of the present analyses, a hospital admission of ≥2 days has been used as the indicator of morbidity.

We have considered all natural, cardiovascular, and respiratory causes for morbidity and mortality from the data sources. International classification of diseases (ICD-10), as proposed by the Word Health Organization (WHO), has been used for the classification of disease-specific mortality in the case of CépiDc data. The sub-classification of J00–J99 has been used for mortality due to respiratory diseases, while I00–I99 is used for mortality due to diseases of the circulatory system. However, the ESPS survey data is questionnaire-based, where individuals were asked about the occurrence of a disease during the past 12 months.

The total number of deaths due to all natural causes, respiratory, and circulatory diseases from the CépiDc data, in the year 2012, amounted to 521,360, 38,092, and 141,295, respectively. The ESPS 2012 survey includes 13,239 individuals who are 15 years or older and participated in the survey. For reasons of privacy, individual data were pooled and averaged at the departmental level and matched to air pollution data. The total number of registered hospital admissions for two days or more has been considered as a measure for morbidity. Likewise, the total number of registered deaths was taken into account as a measure for mortality for each department.

### 2.3. Control Variables

Regarding the control variables, deprivation index, tobacco smoking, and total population were considered for our analysis using the CepiDc data. Due to the unavailability of data for tobacco smoking at the departmental level, we considered lung cancer mortality rates as a proxy for this variable [16,17]. Nevertheless, the analyses using the ESPS survey data is controlled for BMI, tobacco smoking, education, and marital status. The divergence in the use of confounders is due to the respective availability of data in the two different data sources.

### 2.4. Statistical Analysis

The statistical association between air pollution and health outcomes was evaluated by estimating yearly mean data of both air pollution and health data at the departmental level.

The long-term association between air pollutants and health outcomes was modeled using a Poisson regression technique, a generalized linear model form of regression analysis used to model count data and contingency tables, where observed mortality or hospitalization counts were used as the response variable, air-pollution and confounders as explanatory variables, and expected mortality as an offset. Poisson regression assumes the response variable has a Poisson distribution, and that the logarithm of its expected value can be modeled by a linear combination of unknown parameters. This Bayesian model included unstructured and conditional autoregressive (CAR) spatial structure random effects. The statistical model was performed using the Integrated Nested Laplace Approximations (INLA) package of R software (version 3.3.2) [18], which is based on the Besag-York-Mollié model [19]. 

In statistical notation, $M_{i}\sim \mathrm{Poisson}\left(E_{i}\times Y_{i}\right),$ where $M_{i}$ is the observed mortality (or morbidity) counts, $E_{i}$ is the expected mortality (or morbidity) counts, $Y_{i}$ is the standardized mortality (or morbidity) ratio, and i denotes the corresponding French department. Therefore, log(Y_i_) = β_0_ + β_1_X_1i_ + … + β_n_X_ni_ + u_i_ + v_i_, where $X_{1i}\ldots X_{\mathrm{ni}}$ represents the ‘n’ number of independent variables for each department. The terms u_i_ and v_i_ correspond to non-spatial and spatial random effects that capture the heterogeneity between the departments. 

A relative risk (RR) was obtained from the model. The analyses were performed for each separate group of mortality and morbidity using a multi-pollutant approach. A similar statistical design has been used in other studies for investigating the health effects of air pollution [20]. 

For contextual variables at the departmental level, the deprivation index [21] and lung cancer mortality rates as a proxy of smoking habits were taken into account from the CépiDc data. However, in the case of analyses with the ESPS data, the models were adjusted for individual body mass index (BMI; kg/m^2^), tobacco smoking, education, and marital status at the departmental level based on the averages of individual data.

Of note, each of the regression analyses was performed with PM_2.5_ and PM_10_ in separate models (Model 1 and 2), in addition to other pollutants (i.e., NO_2_ and O_3_) as the explanatory variables. This is because the significant association between PM_2.5_ and PM_10_ may lead to the problem of collinearity and multicollinearity and consequently give rise to spurious results if considered in a single model. 

## 3. Results

In 1999–2000, mean levels of PM_10_, PM_2.5_, NO_2_, and O_3_ were within the ranges of 7.93–27.39 µg/m^3^, 4.06–17.10 µg/m^3^, 4.55–46.96 µg/m^3^, and 77.62–111.10 µg/m^3^, respectively, spanning across all departments in France.

Considering the routine mortality data from CepiDc at the department level, exposure to both PM_2.5_ and PM_10_ had a significant 12-year long-term effect on mortality from natural causes [(RR = 1.024; 95% CI = 1.022, 1.026) and (RR = 1.029; 95% CI = 1.027, 1.031)], cardiovascular diseases [(RR = 1.022; 95% CI = 1.015, 1.029) and (RR = 1.047; 95% CI = 1.045, 1.051)], and respiratory diseases [(RR = 1.037; 95% CI = 1.029, 1.031) and (RR = 1.056; 95% CI = 1.043, 1.069)], respectively, as represented in Table 1. NO_2_ was also related marginally to a higher risk for all-cause mortality in both the models, and cardiovascular diseases in Model 2. Additionally, a slightly statistically significant association was seen between O_3_ and all-cause and respiratory mortality in Model 1.

Table 2 represents the long-term effect of air pollutants on all-cause mortality for the ESPS survey data. A prominent impact of particulate matter was observed with a relative risk of 1.032 (95% CI = 1.021, 1.065) for PM_2.5_ and 1.072 (95% CI = 1.052, 1.092) for PM_10_. Additionally, a significant effect was seen for NO_2_ (RR = 1.041; 95% CI = 1.024, 1.058) and O_3_ (RR = 1.018; 95% CI = 1.002, 1.035) in the case of models 2 and 1, respectively. Information on cause-specific deaths were not available from this survey.

Lastly, an association between hospitalizations and air pollutants from the ESPS survey data was estimated, as seen in Table 3. A significant relative risk was estimated for the association between all-cause hospitalizations and NO_2_ [(Model 1: RR = 1.029; 95% CI = 1.002, 1.057) and (Model 2: RR = 1.046; 95% CI = 1.020, 1.074)], PM_2.5_ (Model 1: RR = 1.107; 95% CI = 1.079, 1.136) and PM_10_ (Model 2: RR = 1.099, 95% CI = 1.072, 1.128)]. Further, cardiovascular and respiratory hospitalizations were related to PM_2.5_, PM_10_, and NO_2._ However, no significant relation was observed for all-cause and disease-specific hospital admission and O_3_. 

## 4. Discussion

Considering different nationwide datasets for our analyses in metropolitan France, our results consistently establish the negative impacts of fine particulate matter on morbidity as well as mortality. Our results reaffirm prior epidemiological evidence [2,22] showing the strong link between particulate matter and health outcomes and, in particular, between chronic health effects and exposure to suspended particulate matter, especially PM_2.5_. In line with previous clinical and epidemiological studies [1,5,6,8], a close association of fine particles and gases with long-term effects was observed. As a whole, there are few studies that have dealt with long-term health effects of air pollution. However, most of these studies have considered only limited regions for their analyses [1]. Although a long-term association of PM_2.5_ and mortality risks was seen in the case of a large U.S. cohort study comprising of six states and two cities [2], such a large-scale population- based study is rare for Europe. Therefore, using a nationwide French data to analyze long-term health impacts of air pollution is more informative and representative.

Similar to other prior research [9], our study also provides some evidence of long-term detrimental effects of NO_2_ exposure on diseases and mortality for the data sample from the ESPS survey. However, the association was not found to be as strong as that with particulate matter exposure. This is compatible with a recent review study on the effect of NO_2_ on human health [23] suggesting that there exists moderate evidence of health effects due to long-term NO_2_ exposure, at the limit of 40 µg/m^3^. 

At the same time, contrary to some studies [10], no robust association was found with exposure to O_3._ The lack of consistent outcome is probably due to the use of departmental data instead of individual data. At the same time, the dimension of the sample involved in the ESPS survey may not be adequate to capture the possible effect of O_3_ on mortality and morbidity. In addition, atmospheric O_3_ is present only for some specific months of the year and therefore its effect is diluted when taking into account the entire year. 

In particular, a major strength of our study is the usage of daily concentrations of air pollution at a very small resolution of 2 km. In addition, two sets of population samples were used, where one includes the mortality record of the total population residing in metropolitan France, and the other involves both the morbidity and mortality of individuals from the ESPS survey, which is representative of the general population living in metropolitan France. In spite of the differences in the data samples, the obtained results are concordant. However, in the ESPS survey the statistical power is missing in some cases. This calls for further analyses with more precise health data. An alternative analysis strategy could be the use of morbidity data from one day of hospitalization, as well as the inclusion of emergency room visits. This would lead to more detailed health data. Nevertheless, the same clear trends involving air pollution posing higher risks to morbidity and mortality were seen consistently in both our analyses. 

Furthermore, our analyses were adjusted for both contextual and individual variables, which represent both a strength and a limitation. However, temperature was not considered as a confounder in our analyses because of the lack of representativeness at the departmental level. Additionally, epidemiological studies that take into account temperature as the confounding factor lack a consolidated inference on the adjustment procedure for non-linear and lagged temperature effect, along with the interaction term [24]. Moreover, seasonality was not considered because of prominent variance in temperature at the same period of the year in France, where three types of climate can be found (oceanic, continental, and Mediterranean). In addition, exposure to indoor air pollution, like wood stoves and fireplaces, was not considered in our study, but at the departmental level it is reasonable to think that this was leveled.

A major question about our work involves the significance of the observed impact of air pollution exposure in 1999–2000 on morbidity or mortality in 2012, which could likely be attributed to other years of exposure. However, since our analyses are carried out at the departmental level, the movement of individuals over this time period is insignificant as compared to the total population in each department. In addition, most of the French monitoring stations did not register a significant change for PM_10_ indicators for the period 2002–2011 [25]. Most importantly, since our results are consistent using two different datasets at the departmental level, our findings strongly support the long-term effects of air pollution on mortality and morbidity.

## 5. Conclusions

Our analyses, using nationwide samples, confirm the adverse effects of air pollutants on respiratory, cardiovascular, and all-cause mortality and morbidity. In particular, PM_10_ and PM_2.5_ were found to have the highest effect on health hazard in the long run. The presence of NO_2_ in the atmosphere also affected mortality and morbidity prominently, while the effect of O_3_ was almost negligible in our study. 

It may be noted that although the level of air pollution was found to be higher than the European air quality standards, the level of pollution was still moderate. In spite of less intense levels of pollutants, the air pollution in the study area has evidently impacted the health condition of its population to a significant extent.

## Figures and Tables

**Table 1:** Long-term risk for mortality in 2012 associated with air pollution * exposure in 1999–2000 at the departmental level in Metropolitan France in the CepiDc data.

Model 1	All-Cause	Cardiovascular Diseases	Respiratory Diseases	Model 2	All-Cause	Cardiovascular Diseases	Respiratory Diseases
NO_2_	1.003 (1.003–1.004) **	1.000 (0.999–1.000)	0.994 (0.992–0.995)	NO_2_	1.002 (1.001–1.002)	1.003 (1.003–1.004)	0.998 (0.997–1.000)
PM_2.5_	1.024 (1.022–1.026)	1.022 (1.015–1.029)	1.037 (1.029–1.044)	PM_10_	1.029 (1.027–1.031)	1.047 (1.045-1.051	1.056 (1.043–1.069)
O_3_	1.002 (1.002–1.003)	0.999 (0.997–1.000)	1.009 (1.008–1.009)	O_3_	0.991 (0.990–0.991)	0.993 (0.941-1.049)	1.000 (0.999–1.002)

Note: * According to the CHIMERE dispersion model; ** Relative risk (RR) for 10 µg/m^3^ increase (95% Confidence Interval) of the air pollutant obtained with Poisson regression analysis, controlled at municipal level for deprivation index, lung cancer mortality rates as proxy of tobacco smoking, and total population. Regression analysis was performed with particulate matter of 10 µm or 2.5 µm in diameter or less (PM_2.5_ and PM_10_, respectively) in separate models (Models 1 and 2, respectively). CepiDc: Centre d’Épidémiologie sur les Causes Médicales de Décès/French Epidemiology Centre on Medical Causes of Death.

**Table 2:** Long-term risk for mortality in 2012 associated with air pollution * exposure in 1999–2000 in the French ESPS Survey.

Model 1	Natural Causes	Model 2	Natural Causes
NO_2_	1.012 (0.999–1.027) **	NO_2_	1.041 (1.024–1.058)
PM_2.5_	1.032 (1.021–1.065)	PM_10_	1.072 (1.052–1.092)
O_3_	1.018 (1.002–1.035)	O_3_	0.992 (0.978–1.006)

Note: * According to the CHIMERE dispersion model; ** RR for 10 µg/m^3^ increase (95% Confidence Interval) of the air pollutant obtained with Poisson regression analysis controlled for BMI, tobacco smoking, education, and marital status. Regression analysis was performed with PM_2.5_ and PM_10_ in separate models (Models 1 and 2, respectively). ESPS: Enquête Santé et Protection Sociale/Health, Health Care and Insurance Survey.

**Table 3:** Long-term risk for hospital admissions (≥2 days) in 2012 associated with air pollution. * exposure in 1999–2000 in the French ESPS Survey.

Model 1	All-Cause	Cardiovascular Diseases	Respiratory System Diseases	Model 2	All-Cause	Cardiovascular Diseases	Respiratory System Diseases
NO_2_	1.029	1.084	1.165	NO_2_	1.046	1.071	1.17
	(1.002–1.057) **	(0.917–1.281)	(0.883–1.537)		(1.020–1.074)	(0.876–1.310)	(0.904–1.513)
PM_2.5_	1.107	1.225	1.2	PM_10_	1.099	1.097	1.181
	(1.079–1.136)	(0.967–1.551)	(0.990–1.454)		(1.072–1.128)	(0.899–1.339)	(0.970–1.439)
O_3_	1.008	0.742	0.793	O_3_	0.998	0.919	0.869
	(0.974–1.044)	(0.490–1.123)	(0.473–1.330)		(0.963–1.035)	(0.700–1.208)	(0.645–1.172)

Note: * According to the CHIMERE dispersion model; ** RR for 10 µg/m^3^ increase (95% Confidence Interval) of the air pollutant obtained with Poisson regression analysis controlled for BMI, tobacco smoking, education, and marital status. Regression analysis was performed with PM_2.5_ and PM_10_ in separate models (Models 1 and 2, respectively). ESPS: Enquête Santé et Protection Sociale/Health, Health Care and Insurance Survey.
